# Microbial Neuraminidase Induces a Moderate and Transient Myelin Vacuolation Independent of Complement System Activation

**DOI:** 10.3389/fneur.2017.00078

**Published:** 2017-03-07

**Authors:** Pablo Granados-Durán, María Dolores López-Ávalos, Manuel Cifuentes, Margarita Pérez-Martín, María del Mar Fernández-Arjona, Timothy R. Hughes, Krista Johnson, B. Paul Morgan, Pedro Fernández-Llebrez, Jesús M. Grondona

**Affiliations:** ^1^Laboratorio de Fisiología Animal, Facultad de Ciencias, Departamento de Biología Celular, Genética y Fisiología, Instituto de Investigación Biomédica de Málaga (IBIMA), Universidad de Málaga, Málaga, Spain; ^2^Centro de Investigaciones Biomédicas en Red de Bioingeniería, Biomateriales y Nanomedicina, CIBER BBN, Facultad de Ciencias, Universidad de Málaga, Málaga, Spain; ^3^Division of Infection and Immunity, School of Medicine, Cardiff University, Cardiff, UK; ^4^Alexion Pharmaceuticals Inc., Cheshire, CT, USA

**Keywords:** anti-C5, brain, C5a, C6-deficient rats, complement system, myelin vacuolation, neuraminidase, neuroinflammation

## Abstract

**Aims:**

Some central nervous system pathogens express neuraminidase (NA) on their surfaces. In the rat brain, a single intracerebroventricular (ICV) injection of NA induces myelin vacuolation in axonal tracts. Here, we explore the nature, the time course, and the role of the complement system in this damage.

**Methods:**

The spatiotemporal analysis of myelin vacuolation was performed by optical and electron microscopy. Myelin basic protein-positive area and oligodendrocyte transcription factor (Olig2)-positive cells were quantified in the damaged bundles. Neuronal death in the affected axonal tracts was assessed by Fluoro-Jade B and anti-caspase-3 staining. To evaluate the role of the complement, membrane attack complex (MAC) deposition on damaged bundles was analyzed using anti-C5b9. Rats ICV injected with the anaphylatoxin C5a were studied for myelin damage. In addition, NA-induced vacuolation was studied in rats with different degrees of complement inhibition: normal rats treated with anti-C5-blocking antibody and C6-deficient rats.

**Results:**

The stria medullaris, the optic chiasm, and the fimbria were the most consistently damaged axonal tracts. Vacuolation peaked 7 days after NA injection and reverted by day 15. Olig2+ cell number in the damaged tracts was unaltered, and neurodegeneration associated with myelin alterations was not detected. MAC was absent on damaged axonal tracts, as revealed by C5b9 immunostaining. Rats ICV injected with the anaphylatoxin C5a displayed no myelin injury. When the complement system was experimentally or constitutively inhibited, NA-induced myelin vacuolation was similar to that observed in normal rats.

**Conclusion:**

Microbial NA induces a moderate and transient myelin vacuolation that is not caused either by neuroinflammation or complement system activation.

## Introduction

Myelin disorders range from spontaneous inherited to acquired due to inflammatory processes, autoimmune disorders, infectious processes, and toxic substances (1). Myelin vacuolation is the most frequent pathologic feature of myelin sheaths (1–3), although it should be differentiated from neuronal vacuolation that occurs in some encephalopathies such as bovine spongiform encephalopathy (4). The idea that myelin vacuolation is equivalent to myelin degeneration is not exact, since some pathologies, such as Canavan’s disease, display a conspicuous myelin vacuolation with mild demyelination (1). Myelin vacuolation may occur in at least two ways, the most frequent of which is intramyelinic vacuolation. This is produced when the myelin lamellae split along the intraperiod line, which in turn reopens the extracellular space initially closed during the formation of myelin (5, 6). This mechanism can generate vacuoles (filled with unstainable material) at multiple levels within the myelin sheath and has been associated with fluid accumulation in the nervous system parenchyma (6). Vacuolation can be caused by inherited conditions (7), as is the case in Canavan’s disease (8), or by the effects of toxins (9–12). The other mechanism of myelin vacuolation occurs when the lamellae split at the major dense line, thus reopening the intracellular space of the oligodendrocyte or the Schwann cell (6). Whatever the etiology of vacuole formation, the progression from myelin vacuolation to demyelination may depend on whether the primary defect or target is the myelin sheath or the myelinating cells, and on the time of exposure to the inducing agent (1).

Gangliosides are sialic acid-containing glycosphingolipids located in the outer leaflet of the plasma membrane, which bear a glycan moiety extending out into the extracellular side (13). The myelin-associated glycoprotein (MAG), a transmembrane glycoprotein specific for myelinating cell membranes (14), specifically binds to axonal gangliosides through the terminal α2,3-linked sialic acid (15–17). Affinity studies showed that MAG binds preferentially to GD1a and GT1b (15), the latter present in white matter. In the central nervous system (CNS), MAG is located only on the innermost wrap of myelin, in direct contact with the axonal surface (18); this location has endorsed its involvement in axon–myelin recognition (19).

Neuraminidase (NA) is an exo-glucosidase that removes terminal sialic acid from glycan chains, preferably those joined by α2,3 linkages (20). It has been reported that some of the viruses associated with subsequent myelin disorders contain NA in the coat. A single intracerebroventricular (ICV) injection of *Clostridium perfringens* NA within the lateral ventricle (LV) of rats induces a partial ependymal denudation that affects mainly the ipsilateral ventricle with minimal spread to the contralateral and third ventricles (21–23). Recently, the neuroinflammatory events triggered by NA ICV injection have been described (24), and the participation of the complement system in ependymal cell death by NA has been demonstrated (25). However, although the vacuolation of some axonal tracts has been described (23, 24), a detailed study of such alterations in the axonal tracts had not been performed.

The role of the complement system in some demyelinating disorders is an issue of debate and the precise function of complement in such processes remains elusive (26). So, while some reports speak about a protective role of complement in demyelinating processes (27, 28), others point to its involvement in immune-mediated myelin diseases (29–34), or even its lack of participation (35). In neuromyelitis optica, an autoimmune disease of the CNS displaying intramyelinic edema and tissue vacuolation, the complement activation is observed in damaged tissue (36). On the other hand, several facts suggest the participation of the complement system in NA-induced myelin vacuolation: (i) sialic acid removal from human tumor cells and red blood cells makes them susceptible to alternative pathway activation and formation of the membrane attack complex (MAC) (37, 38); (ii) NA can directly activate the alternative complement pathway (39); and (iii) the presence of NA within the cerebral ventricles triggers the activation of the complement system which contributes to ependymal disruption (25).

The present report describes the spatial distribution and the time course of the myelin vacuolation induced by intraventricular NA. Damage to myelin sheaths, oligodendrocyte cells, and neurons were assessed. Furthermore, the role of the complement system was investigated by injecting the anaphylatoxin C5a in the ventricles of normal rats, and also by the administration of NA to two rat models in which the complement system is disturbed.

## Materials and Methods

### Animals

Adult male Wistar rats (7 weeks old) with 250–300 g body weight (Charles River Laboratories, Barcelona, Spain) were used in this study. In addition, a strain of rats deficient in the C6 component of complement, where the formation of the MAC is blocked (32, 40), was used as well. Between five and six animals were used for each time or experimental situation involving NA injection (unless otherwise indicated), while only three animals were used as control rats. All animals were housed under a 12-h light/dark cycle with food and water available *ad libitum*. Animal procedures were performed according to the European Union (86/609/EEC) and Spanish (RD 1201/2005) legislations. Animal care and experimental procedures were approved by the Animal Experimentation Ethics Committee of the Universidad de Málaga (ref. number 2012-0013).

### ICV Injection

Animals were anesthetized with 2,2,2-tribromoethanol (0.2 g/kg bw, Fluka Chemika) and positioned in a stereotaxic frame. NA from *C. perfringens* (Roche Diagnostics, 11 585 886 001) dissolved in 0.9% sterile saline (25 mU/μl) was administrated by stereotaxic surgery into the right lateral cerebral ventricle. The coordinates from Bregma used were antero-posterior −0.5 mm, medio-lateral −1.4 mm, and dorso-ventral −3.5 mm. A single dose of 500 mU (20 μl) of NA was injected with the aid of a pump at a rate of 2 μl/min over 10 min. Some animals were sacrificed immediately after NA injection (time 0 h), and the rest were recovered from anesthesia and sacrificed at 2 h, 4 h, 12 h, 24 h, 2 days, 4 days, 7 days, 15 days, and 30 days after the administration of NA. For each time, three control rats were ICV injected with saline and processed. In order to know whether the neuroinflammatory reaction itself could induce the myelin vacuolation, four rats were ICV injected with the anaphylatoxin C5a (5 μg/20 μl; Sigma-Aldrich ref. C5788), which generates a severe neuroinflammation (41). These animals were sacrificed 24 h after the C5a injection and processed for histological studies. Although similar experiments have been carried out in previous studies (41), the location of vacuolization at very specific sites, as it is in our NA-injected rats, could have gone unnoticed.

### Inhibition of the Complement Component C5

In order to obtain both systemic and ventricular inhibition of complement component C5, a strategy consisting of the injection of a blocking anti-rat C5 monoclonal antibody [C5-inh; clone 18A10, Alexion Pharmaceutical, Inc. (42, 43)] was employed. Four animals were used in this experiment. The regimen of anti-C5 administration was as previously described (44); an initial intravenous injection (20 mg/kg bw) was followed 6 h later by an intraperitoneal injection (10 mg/kg bw). According to the published data (44), this protocol resulted in a 50–60% inhibition of C5 activity for up to 12 h after the first injection, with the highest inhibition occurring about 1 h after the second injection. At this time of maximal systemic inhibition, the antibody was also injected ICV to guarantee the inhibition of C5 within the ventricular system. In those animals treated with anti-C5 and NA, both were ICV injected simultaneously (20 μl of a mixture of NA 25 mU/μl and C5-inh 0.38 μg/μl) following the same parameters outlined above. After 7 days of this treatment, rats were perfused and the brains processed for histological and immunocytochemical studies.

### Histological Procedures

Animals were anesthetized with 2,2,2-tribromoethanol and transcardially perfused with saline followed by Bouin’s fixative (45). Brains were removed and immersed in the same fixative for 24 h, and later embedded in paraffin wax. Seven-micrometer sections were obtained from each brain and mounted on poly-l-lysine-treated slides. Series of sections along the brain region of interest (from Bregma −0.3 to −1.2 mm approximately) were obtained. Hematoxylin–eosin staining was applied to counterstain the sections.

### Immunohistochemistry

Immunohistochemistry was carried out on deparaffinized tissue sections using the immunoperoxidase technique. The primary antibodies used were as follows: rabbit anti-rat C5b-9 (1:200, Abcam, ab55811); rabbit anti-rat C9 (1:100, provided by Prof. B. P. Morgan, Cardiff University); rabbit anti-myelin basic protein (MBP) (1:500, Zymed, 18-0038); goat anti-Olig2 (1:100, RyD System, AF2418); and mouse anti-caspase-3 (1:100, Thermo, MS1123R7). The secondary antibodies used were as follows: biotinylated goat anti-rabbit IgG (H + L) (1:1,000, Pierce, 31820); biotinylated horse anti-goat IgG (H + L) (1:1,000, Vector, BA9500). For the mouse antibody against caspase-3, the Ultravision Quanto HRP system (Thermo Fisher Scientific, TL060QHD) was used. All antibodies were diluted with PBT buffer (0.3% bovine serum albumin, 0.3% Triton X-100 in PBS pH 7.3). Primary antibodies were incubated overnight at room temperature (RT). Secondary antibodies were incubated for 60 min at RT. Negative controls for the immunostaining consisted of equivalent sections subjected to the same protocol but omitting the primary antibody.

Prior to the overnight incubation with the primary antibody, the sections stained with the immunoperoxidase procedure were incubated in 3% hydrogen peroxide and 10% methanol in PB 0.1 M to quench endogenous peroxidase activity. In such sections, biotinylated secondary antibody was used, along with the avidin–biotin complex (ABC) amplification method. The ABC reagent was prepared according to the manufacturer’s instructions (Thermo Fisher Scientific) and incubated for 30 min. Peroxidase activity was revealed with 0.05% DAB and 0.03% hydrogen peroxide. All the incubations were performed in a moist chamber at RT. Hematoxylin–eosin or only hematoxylin was used to counterstain some immunostained sections; however, it was not used with a weak immunostaining (as anti-C5b9) or to quantify oligodendrocytes.

### Electron Microscopy

Three ICV NA-injected rats were perfused with Karnovsky’s fixative (46). Once extracted, the brains were kept in the same fixative overnight at 4°C. Vibratome sections (200 μm thick) were used to obtain pieces (3 mm × 3 mm) of different brain areas. These were post-fixed in 2% osmium tetroxide (in 0.1 M phosphate buffer, 1 h at 4°C), dehydrated in graded alcohol series, and embedded in Araldite resin (Grade 502, Sigma-Aldrich, ref. AR502). Ultrathin sections (50–70 nm thick) were obtained with an ultramicrotome (Leica, EM UC7) using a diamond knife (Diatome, Ultra 45°). Sections were contrast stained with lead citrate and uranyl acetate, and analyzed using a transmission electron microscope (Geol JEM-1400, 80 kV). Electron micrographs were taken with a digital camera (Gatan, ref. ES1000W). Sectioning and ultrathin observation were performed at the electron microscopy core facility at Universidad de Málaga.

### Fluoro-Jade B Staining

Fluoro-Jade B is a polyanionic fluorescein derivative that specifically binds to degenerating neurons (47), regardless of whether the death is through necrosis or apoptosis. This compound has an excitation peak at 480 nm and an emission peak at 525 nm. Sections from five rats (12 h after NA treatment) were deparaffinized and hydrated in alcohols in the conventional manner, but including a 5-min step in 80% ethanol with 1% NaOH. Then, the sections were incubated in 0.06% potassium permanganate solution (10 min), washed with distilled water (2 min), and then incubated with a 0.0004% solution of Fluoro-Jade B in 0.1 M acetic acid solution (20 min). The sections were then washed with distilled water (3× 1 min) and air dried 10 min, and further dried on a hotplate at 50°C for 10 min. Once dried, they were immersed in xylene for 5 min and mounted with Eukitt^®^. This staining was applied to Bouin’s-fixed paraffin sections. Sections of the cortical region directly damaged by the injection needle were used as positive control.

### Quantification of Myelin Vacuolation

In order to assess the time course of myelin vacuolation in NA-injected rats, MBP immunoreactivity was quantified in ipsilateral and contralateral areas of animals sacrificed at different times postinjection (0 h, 1 day, 2 days, 4 days, 7 days, 15 days, and 30 days). Three sections from each animal (*n* = 4) (Bregma −0.92, −0.95, and −0.99 mm approximately) were chosen for the quantification. In any case, the selected sections were close to the foramen of Monro but far enough from the site of injection as to avoid areas of surgically damaged tissue. Such sections contained areas of interest including the stria medullaris (SM) and the optic chiasm. However, the fimbria was not analyzed because it is too close to the injection site and could be affected by the damage of the needle. From each histological section, two photographs were taken: one in the SM and another in the optic chiasm, both in the ipsilateral side. Photographs were always taken under the same conditions. Pictures were then processed using the image analysis software Visilog 6.3 (Noesis, France) to determine the proportion of MBP-positive area per image. Afterward, the mean value was obtained from the three sections studied from each animal, which was considered the percentage of MBP-stained area of that animal. A total of five animals were analyzed at each postinjection time studied. By using such methodology, the higher vacuolation, the less percentage of MBP-stained area.

### Quantification of Oligodendrocytes

A quantitative method was used to determine whether oligodendrocytes were damaged in the NA-treated rats. The oligodendrocyte immunocytochemical marker Olig2 (48) was applied on the same animals used for the semiquantification of MBP. From each animal (*n* = 4), four sections (spaced 70 μm apart) within the region of the foramen of Monro (between rostrocaudal Bregma levels −0.85 and −1.13 mm) were employed. Two photographs, one from the SM and other from the optic chiasm, were taken from each section using the 40× objective lens. The Olig2-positive cells were counted and referred to the quantified area. The average value was obtained from the four sections studied from each animal. Five animals were analyzed for each postinjection time studied.

### Statistical Analysis

Differences between times postinjection of NA were evaluated by ANOVA (significance level of 0.05), followed by a Tukey *post hoc* test with a level of significance of 0.05. The Shapiro–Wilk test was used to ensure that the data met the criteria of normality to allow the use of parametric statistics. The Levene’s test was performed before the ANOVA to test the equality of variances. The values measured at time 0 were considered the baseline. The software SPSS^®^ Statistics 20 (IBM^®^) was used for this analysis.

## Results

### Spatial Distribution and Time Course of Myelin Vacuolation in NA-Injected Rats

**Figure 1:**
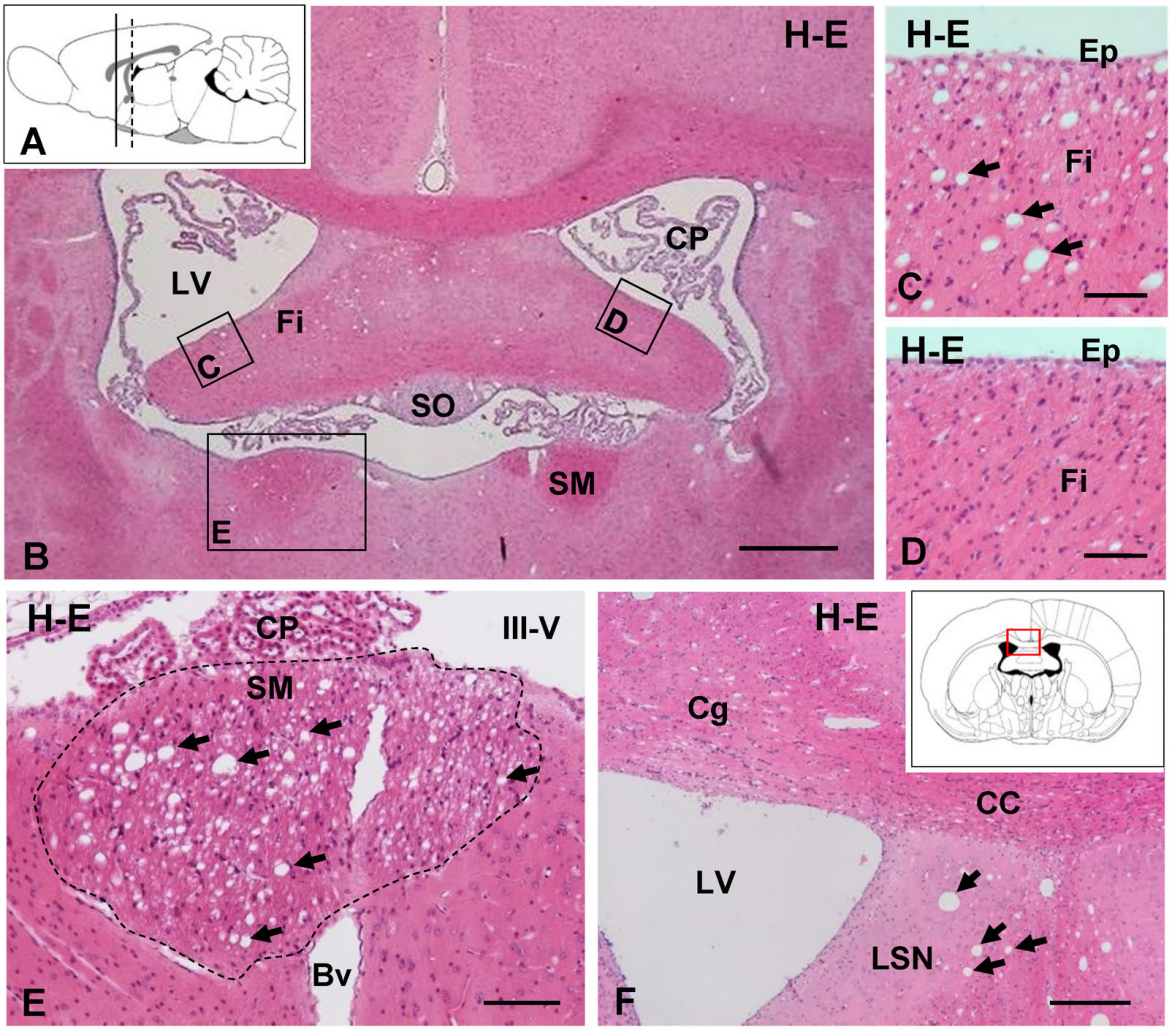
**Intracerebroventricular (ICV) injection of neuraminidase (NA) induces vacuolated lesions in axonal bundles**. **(A)** Sagittal scheme of the rat brain illustrating the coronal levels studied: continuous line [AP = −1.0 mm; **(B–D)**] and discontinuous line [AP = −0.3 mm; **(E,F)**]. **(B)** Coronal section of a rat brain 7 days after ICV injection of NA. **(C)** Detail of the fimbria (Fi) in the injected ventricle with vacuolar lesions (arrows). **(D)** Detail of the contralateral fimbria without alterations. **(E)** Detail of the SM (area outlined with the dotted line) presenting abundant vacuoles (arrows). **(F)** Ipsilateral ventricular region with vacuolar lesions (arrows) in the septum. Inset in panel **(F)**: scheme of a coronal brain section framing the area showed in panel **(F)**. All sections were stained with hematoxylin and eosin (H–E). Bv, blood vessel; CC, corpus callosum; Cg, cingulated; CP, choroid plexus; Ep, ependymal; III-V, third ventricle; LSN, lateral septal nucleus; LV, lateral ventricle; SM, stria medullaris; SO, subfornical organ. Magnification bar = 400 μm in panel **(B)**, 60 μm in panels **(C–E)**, and 200 μm in panel **(F)**.

In brain sections stained with hematoxylin–eosin, vacuolar or spongiform areas were evident in different brain regions 7 days after the ICV injection of NA, although occasionally such alterations could be observed as early as 48 h. The affected zones corresponded to myelinated axonal tracts lying near the injected ventricle, such as the fimbria–fornix (Figures 1B–D) and the SM (Figure 1E), as well as more distant myelinated tracts, including the optic chiasm (Figure 5G). These tracts were consistently affected in all NA-treated rats, and the vacuoles correspond to the space generated adaxonally in some myelinated axons, as could be observed by electron microscopy (see below). Myelin vacuolation was mostly ipsilateral to the injection site, although as it extended along the tract it was sometimes possible to observe mild vacuolization in decussated tracts at the contralateral side.

**Figure 2:**
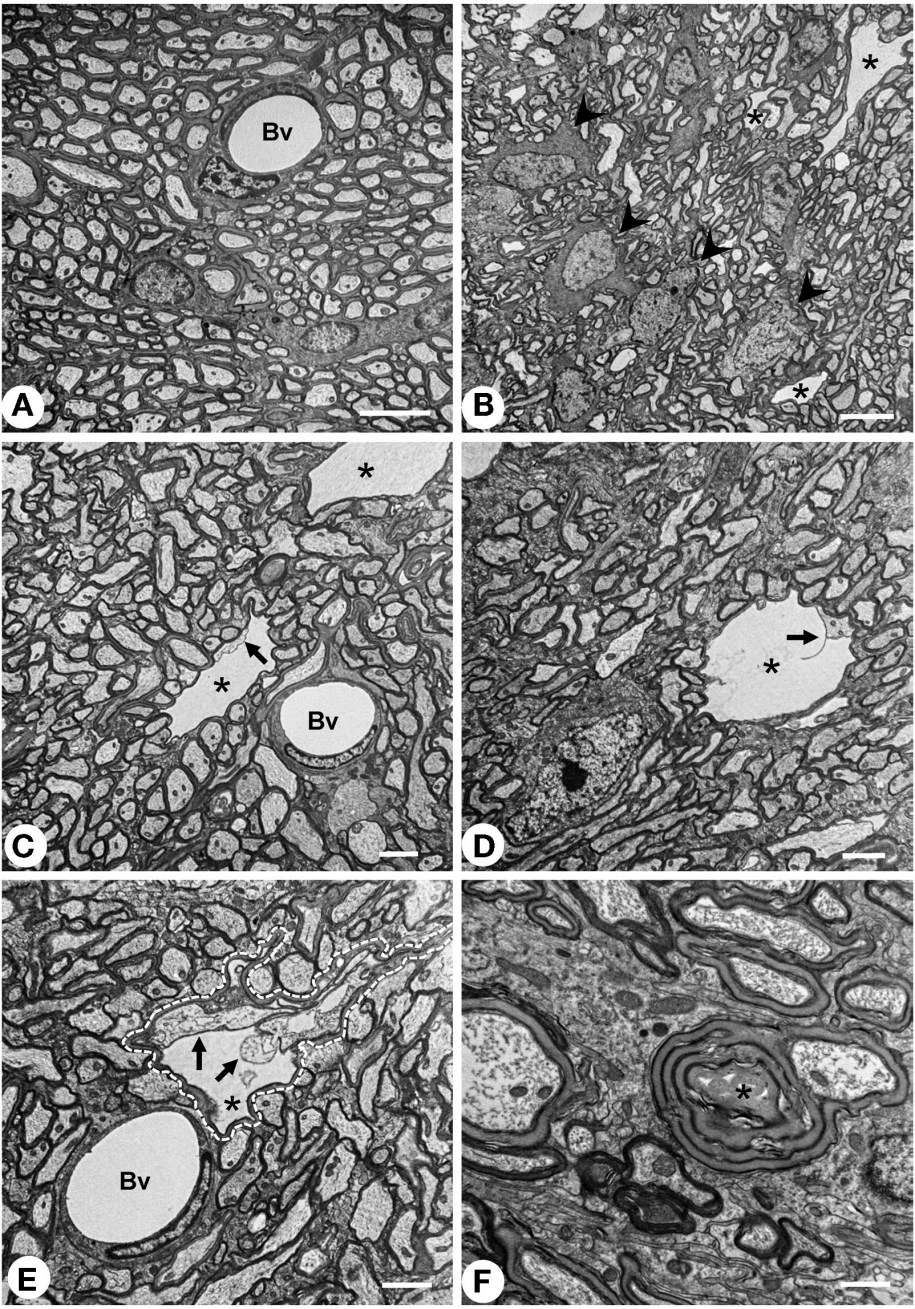
**Transmission electron microscopy of the myelin vacuolar lesions**. **(A)** Stria medullaris (SM) from a normal rat, where myelinated axons display uniform and well packed myelin sheaths. **(B–E)** SM from neuraminidase-injected rats 7 days postinjection. In these animals, wide empty spaces (asterisk) that are surrounded by a myelin sheath can be observed. Such cavities are largely free of electrodense material, and an axon can be found inside (arrows). Such spaces most probably correspond to the vacuoles described by conventional histology. One of these cavities is outlined with a dashed line in panel **(E)**. Oligodendrocyte cells [arrowheads in panel **(B)**] display an apparently normal morphology. **(F)** Abnormal myelinated axon with a disorganized myelin sheath (asterisk). Bv, blood vessel. Magnification bar = 5 μm in panels **(A,B)**, 2 μm in panels **(C–E)**, and 1 μm in panel **(F)**.

Vacuolated areas were analyzed by transmission electron microscopy in order to explore the ultrastructural features of the holes. While control animals displayed axons with uniform and regular myelin sheaths, in the NA-injected rats some dilated and irregularly shaped myelin sheath were observed in transverse sections of the SM (Figure 2E, dotted line encloses one myelinated axon). Such dilated myelin sheath displayed an inner cavity, probably corresponding to the vacuoles observed by conventional histological techniques. Despite its larger diameter, the dilated myelin sheaths themselves were apparently similar to that observed in non-affected axons, or in axons from untreated rats. Within the vacuoles, an axon was consistently found, partially in contact with the myelin sheath (arrows in Figures 2C–E). Therefore, the cavity seems to arise from the space between the axonal surface and the innermost myelin wrap. Rarely axons showing a disorganized myelin sheath were found (asterisk in Figure 2F). These images are compatible with a partial disruption of the interaction between the innermost lamella of myelin and the axonal membrane.

**Figure 3:**
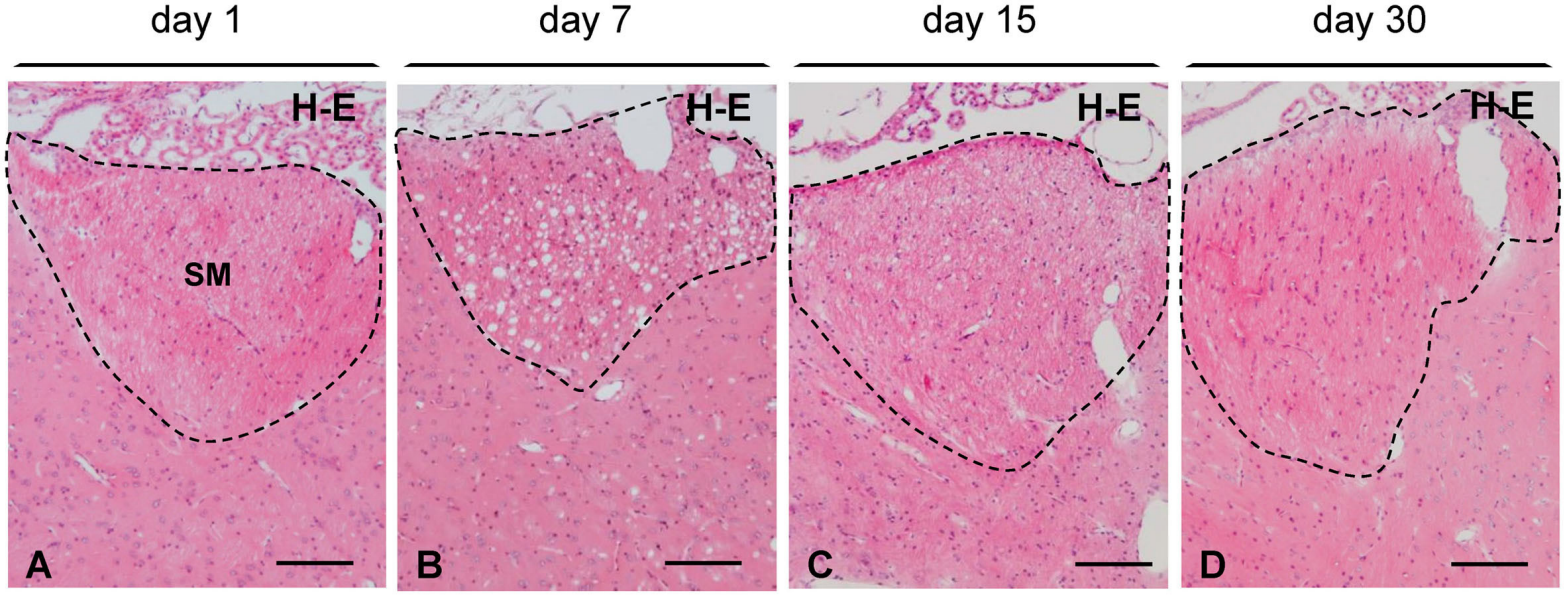
**Time course of myelin vacuolation in the SM after a single intracerebroventricular injection of neuraminidase**. **(A)** One day after the treatment no vacuolation is observed. **(B)** At day 7, the density of vacuoles reaches a peak. **(C)** At day 15, most vacuoles have disappeared. **(D)** After 30 days, the SM (and other axonal bundles) recovers its normal appearance. All sections were stained with hematoxylin and eosin (H–E). SM, stria medullaris. Magnification bar = 100 μm in panels **(A–D)**.

Concerning the time course, myelin vacuolation reached a peak between 4 and 7 days postinjection of NA (compare Figure 3A with Figure 3B). However, from day 15 on, the number and the size of the holes were significantly reduced (Figures 3C,D), and 30 days after the NA injection, they were completely absent (Figure 3D). After 30 days, the SM and the fornix as well as the farthest myelinated tracts (e.g., the optic chiasm) were undistinguishable from those of non-injected rats, with a uniform hematoxylin–eosin staining and the absence of cavities.

### Immunohistochemical Analysis of the Myelin Vacuolation

**Figure 4:**
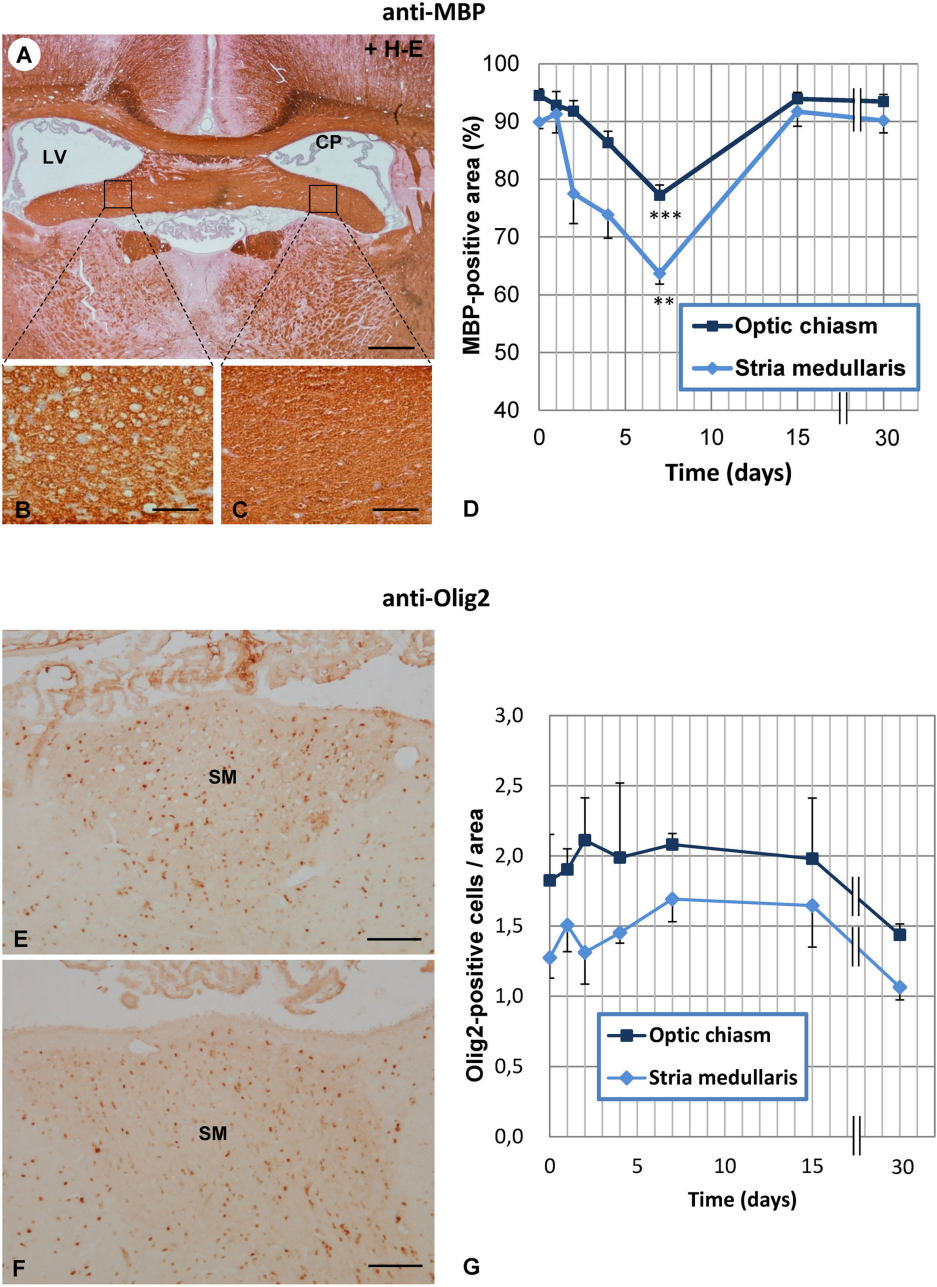
**Time course of vacuolation and oligodendrocyte density in the SM and optic chiasm of neuraminidase (NA)-injected rats**. **(A)** Transverse section at the level of the foramina of Monro immunostained with anti-myelin basic protein (MBP). **(B,C)** The NA-injected ipsilateral fimbria **(B)** displays numerous vacuoles compared with the equivalent contralateral area **(C)**. **(D)** The MBP-positive area was quantified in the ipsilateral SM and optic chiasm at several times after NA injection. The lowest MBP-positive area (corresponding to the highest level of vacuolation) was measured at 7 days postinjection in both the SM and the optic chiasm. **(E,F)** The ipsilateral **(E)** and contralateral **(F)** SM were immunostained with anti-Olig2 to quantify the number of oligodendrocytes in these axonal tracts. **(G)** Olig2-positive cells were counted in the ipsilateral side of both the SM and in the optic chiasm. No significant differences were found between the different postinjection times analyzed. The graph represents the mean ± SEM. Values at different times were compared by one-way ANOVA and a Tukey *post hoc* analysis. **P < 0.01; ***P < 0.001 compared to time 0 days. Sections in panels **(A–C)** were counterstained with hematoxylin and eosin (H–E). CP, choroid plexus; LV, lateral ventricle; SM, stria medullaris. Magnification bar = 400 μm in panel **(A)**, 60 μm in panel **(B)**, and 100 μm in panels **(E,F)**.

The percentage of MBP-positive area was estimated at various postinjection times (1, 2, 4, 7, 15, and 30 days) in two consistently vacuolated areas: the SM and the optic chiasm (Figure 4D). From the second day after treatment, MBP-positive area tended to decrease in the two regions studied (Figure 4D), and at day 7, such reduction was significant compared to control MBP levels (0 days). From day 7 on, MBP-positive area progressively recovered, and by day 15, it was similar to that of non-treated rats (Figure 4D).

Olig2-positive cells were quantified in the SM and the optic chiasm at different times postinjection of NA (Figure 4G), but no significant variations were found at any of the times analyzed, not even at 7 days when vacuolation reached a peak. These results indicate that the number of oligodendrocytes was not modified by the treatment.

### Cell Death Analysis in Vacuolated Axonal Bundles

**Figure 5:**
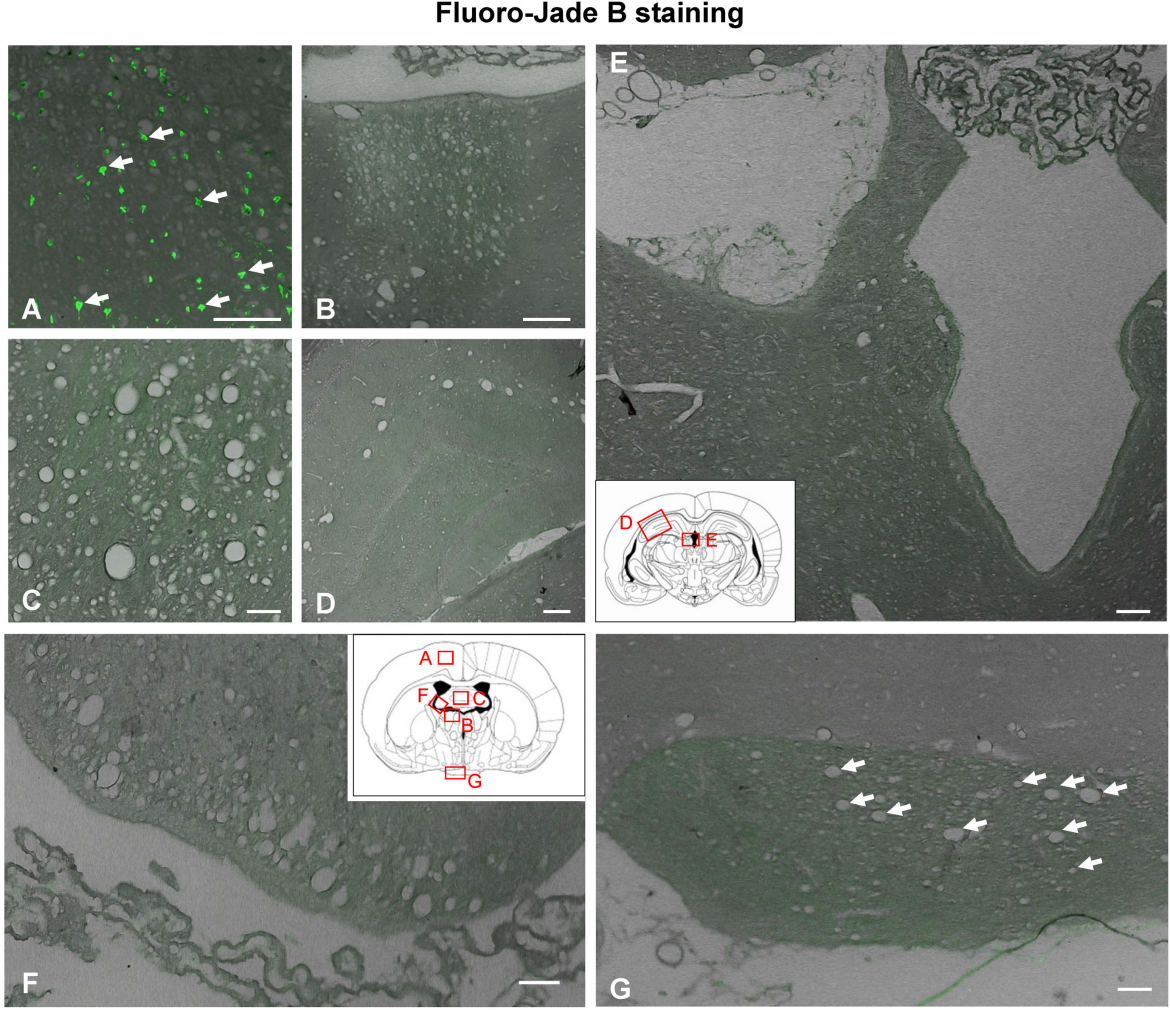
**Neuronal degeneration determined by Fluoro-Jade B staining 1 day after intracerebroventricular (ICV) injection of neuraminidase**. **(A)** Neuronal degeneration displaying green fluorescence (arrows) was revealed by Fluoro-Jade B in the cerebral cortex at the site of ICV injection, a region used as a positive control of the technique. **(B–G)** However, no fluorescent cells were detected in any area of the brain analyzed: **(B)** stria medullaris, **(C)** septum, **(D)** hippocampus, **(E)** cerebral aqueduct, **(F)** fimbria of the hippocampus, and **(G)** optic chiasm. Arrows in panel **(G)** point to vacuoles in the optic chiasm. Insets in panels **(E,F)**: schemes of coronal brain sections framing the areas shown in the photographs. Magnification bar = 100 μm in panels **(A,B)**, 75 μm in panel **(C)**, 200 μm in panel **(D)**, 150 μm in panel **(E)**, 70 μm in panel **(F)**, and 50 μm in panel **(G)**.

**Figure 6:**
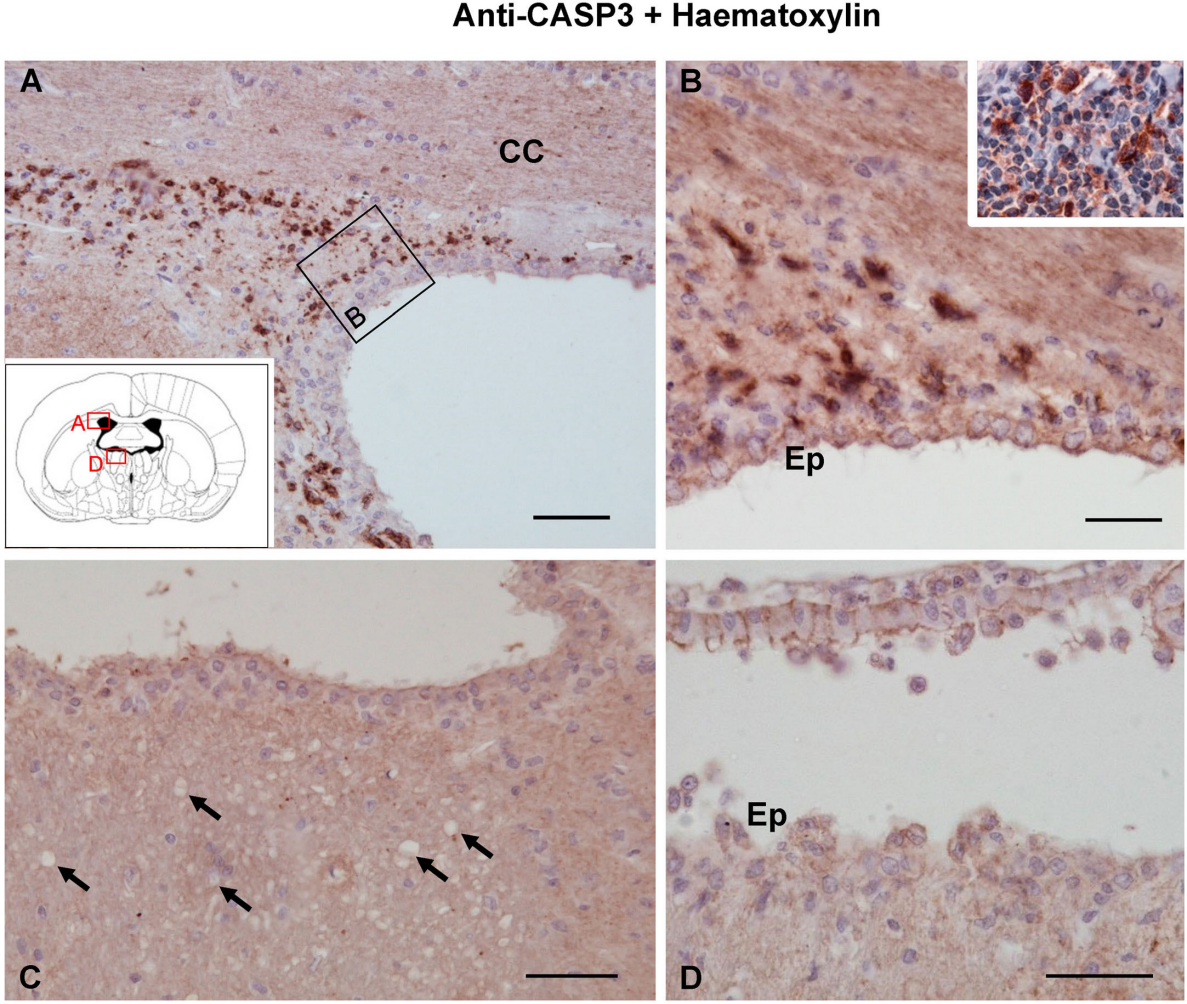
**Apoptosis detected by caspase-3 immunostaining 1 day after the intracerebroventricular injection of neuraminidase (NA)**. **(A,B)** Caspase-3-positive cells were found in the lateral ventricle subventricular zone, where apoptosis occurs as part of the neurogenesis process. Staining was also evident in the palatine tonsil [inset in panel **(B)**] used as a positive control. **(C,D)** No caspase-3-positive cells could be detected either in the stria medullaris (SM) or in the ventricular wall, where ependymal cells are damaged by NA. Ependymal cells were negative since they die by necrosis. Arrows point to vacuoles in SM. All sections were counterstained with hematoxylin. CC, corpus callosum; Ep, ependyma. Magnification bar = 50 μm in panel **(A)**, 30 μm in panel **(B)**, and 50 μm in panels **(C,D)**.

Fluoro-Jade B binds specifically to degenerating neurons undergoing necrosis or apoptosis. As a positive control, we used sections of the dorsal frontal cortex containing neurons mechanically damaged due to the injection procedure (Figure 5A). While these control neurons displayed the fluorescent staining (Figure 5A), no other neuronal degeneration event was detected at any time analyzed or in any of the regions studied, which included the myelinated tracts consistently affected by vacuolation (Figures 5B,C,F,G) and those areas projecting axons to the vacuolated bundles, such as the habenula or the hippocampus (Figures 5D,E). CASP3-positive cells were found in the subventricular neurogenic zone in all animals (Figures 6A,B), highlighting the normal apoptosis that occurs in the neurogenic niche. However, no other CASP3-positive cell was detected in any of the regions studied, i.e., the damaged ependymal epithelium of the injected ventricle (Figure 6D), and myelinated tracts typically affected by degeneration, such as the SM (Figure 6C). Ependymal cells were negative since they die by necrosis.

### Role of the Complement System in the Vacuolation Process

To unravel whether activation of complement might be involved in the vacuolation process observed in NA-injected rats, several experimental approaches were performed: (i) immunolocalization of the MAC on vacuolated bundles, (ii) ICV injection of the anaphylatoxin C5a in normal rats, and (iii) ICV injection of NA in two different rat models in which the complement system is inhibited.

**Figure 7:**
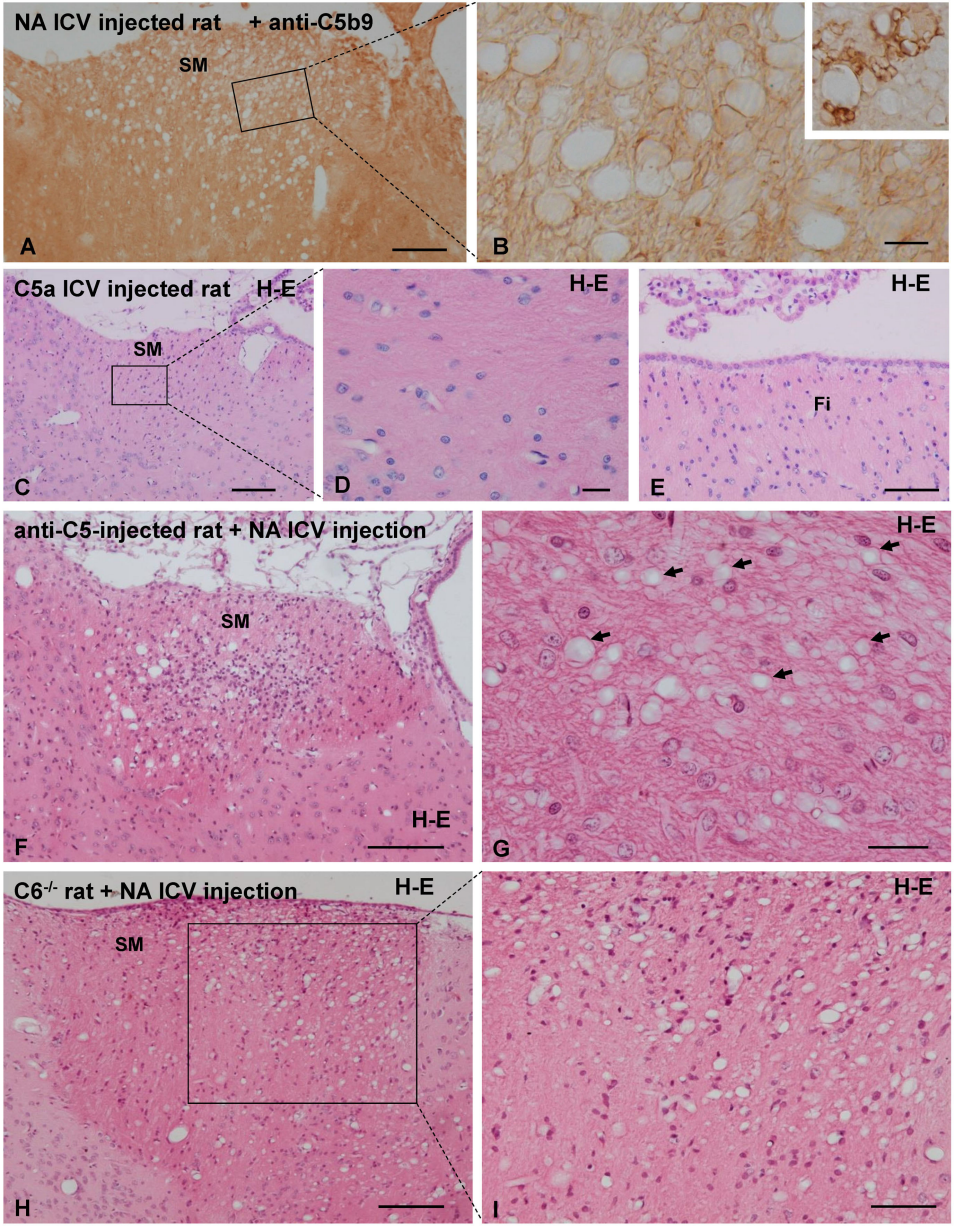
**Analysis of the role of the complement system in neuraminidase (NA)-induced myelin vacuolation**. **(A,B)** The formation of membrane attack complex deposits was evaluated by immunohistochemistry using a specific anti-C5b9 antibody. Labeling with this marker was evident in swollen lymph node used a positive control [inset in panel **(B)**] but completely absent in vacuolated SM. Ependymal cells were absent in this section due to NA treatment, and therefore, no labeling with anti-C5b9 was observed. **(B)** Higher magnification of an equivalent area squared in panel **(A)**. **(C–E)** Rats were intracerebroventricular (ICV) injected with the anaphylatoxin C5a to generate a neuroinflammation process similar to that induced by NA. Seven days after C5a injection, the axonal bundles usually affected by NA injection, that is the SM **(C,D)** and the fimbria **(E)**, displayed no abnormalities. **(F,G)** The complement system was inhibited by systemic and ICV injection of a functional monoclonal antibody against C5. Animals treated for complement inhibition were then subjected to a single ICV injection of NA. Myelin vacuolation observed in the SM was similar in anti-C5-treated and non-treated rats, indicating that complement blockade does not affect the NA-induced vacuolation process [arrows in panel **(G)** point to the vacuoles]. **(H,I)** Rats lacking the C6 component of the complement system were injected with NA to evaluate myelin vacuolation. The vacuolar lesions observed in the SM of these rats were similar to those of wild-type rats (not shown). Sections in panels **(C–I)** were stained with hematoxylin and eosin (H–E). SM, stria medullaris. Magnification bar = 200 μm in panel **(A)**, 15 μm in panel **(B)**, 200 μm in panel **(C)**, 20 μm in panel **(D)**, 50 μm in panel **(E)**, 200 μm in panel **(F)**, 25 μm in panel **(G)**, 200 μm in panel **(H)**, and 50 μm in panel **(I)**.

None of the antibodies against MAC (anti-C5b9 and anti C9) revealed a positive labeling in any of the vacuolated axonal bundles studied (Figures 7A,C), thus suggesting the absence of MAC deposition on vacuolated tracts. Swollen lymph nodes were used as a positive control for anti-C5b9 (inset in Figure 7B).

In spite of the conspicuous neuroinflammation caused by the ICV injection of C5a, the tracts that regularly suffer vacuolation upon NA injection (i.e., SM, fimbria–fornix, and optic chiasm) appeared completely normal in C5a-injected rats.

In animals under MAC inhibition with anti-C5, the myelin vacuolization was similar to that observed in “non-inhibited” rats (Figures 7F,G). In C6^−/−^ rats, the ICV injection of NA induces a vacuolation similar to that observed in normal rats injected with NA (Figures 7H,I). Therefore, the absence of MAC does not prevent the myelin vacuolation induced by NA. It could be hence concluded that complement MAC is not involved in the etiology of myelin vacuolation in this particular model.

## Discussion

In the previous studies, a single ICV injection of a high dose of NA in the LV of rats resulted in the prompt and extensive death of ependymal cells, followed by an inflammatory reaction, myelin vacuolation, and long-term hydrocephalus (23). These severe effects were lessened by the administration of lower doses of NA, which resulted in neuroinflammation, a partial loss of ependymal cells, and myelin vacuolation (24). Recently, the implication of the complement system in NA-induced ependymal death has been reported (25). Here, we examined the noticeable alterations provoked by NA in some axonal tracts and the possible implication of the complement system.

The administration of NA in the LV elicited damage in multiple axonal tracts; those consistently affected were the fimbria–fornix complex, the SM, and the optic chiasm, all them on the ipsilateral side. Several factors could account for the recurrent damage of these particular axonal tracts: (i) their proximity to the ventricular surface or to the pial surface; (ii) their vicinity to the site of the ICV injection, and (iii) the way cerebrospinal fluid (CSF) distributes NA within the ventricular system. NA injected in the LVs reaches the brain parenchyma up to about 400 μm distance from the ependymal surface or the pial surface (22, 49). Therefore, NA may directly act on the axonal tracts close to the site of injection.

The affected axonal tracts displayed numerous vacuoles between days 4 and 7 after NA injection, when vacuolation reaches its peak (the MBP-stained area is the lowest). However, several facts suggest that the alterations triggering myelin vacuolation may be only moderate: (i) most of the vacuoles disappear 15 days after the NA injection suggesting the repair of the damaged axonal bundles; (ii) transmission electron microscopy showed that the vacuoles appear between the axonal membrane and the innermost wrap of myelin (adaxonal vacuolation), while an apparently normal myelin sheath was observed; (iii) oligodendrocyte number was not affected in the vacuolated axonal bundles; and (iv) neither neuronal degeneration nor neuronal apoptosis was observed in neuronal nuclei related to the damaged axonal bundles.

Concerning the etiology of the vacuoles, it is well established that myelin vacuolation can occur by at least two ways: (i) split along the intraperiod line, called intramyelinic vacuolation, which opens the extracellular space between myelin lamellae or between the axon membrane and the myelinating cells, and (ii) split of lamellae at the major dense line, which opens the intracellular space of the oligodendrocyte or the Schwann cell (5, 6). Intramyelinic vacuolation results in vacuoles located at multiple levels within the myelin sheath or between the axonal membrane and the innermost wrap of myelin, and it has been associated with the accumulation of fluid that is non-stainable by conventional histological methods (6). The vacuolation triggered by NA likely represents intramyelinic vacuolation, since the vacuoles are preferentially located between the myelin and the axonal membrane.

Myelin-associated glycoprotein is a transmembrane glycoprotein member of the siglec family of sialic acid-binding proteins (50, 51), which is specific for myelinating cell membranes (14). MAG is only present in the innermost wrap of myelin (18), suggesting a functional role in axon–myelin interactions (52). On the other hand, complex gangliosides displaying terminal α2,3-linked sialic acid (GT1b and GD1a) are present in the neuronal membranes (53), and GT1b is the only one which both strongly binds to MAG and is present in white matter (15, 53). Pretreatment of gangliosides with NA abolished the adhesion of MAG to either GT1b or GQ1b alpha (15, 54), indicating that ganglioside–MAG interaction is dependent on terminal sialic acid (55). Therefore, we hypothesize that the sialidase activity of NA may disrupt MAG–ganglioside recognition and hence the axon–myelin interaction, thereby promoting the onset of the adaxonal vacuolation. NA cytotoxic effects cannot be ruled out in the process. Such mechanism of vacuolation should give rise to myelin abnormalities similar to those described in *Mag*-null or *B4galnt1*-null mutant mice. *Mag*-null mice display delayed myelination and frequent myelin structural abnormalities (56, 57). Similar alterations were found in *B4galnt1*-null mice, which are considered completely devoid of complex gangliosides (58, 59). However, myelin vacuolation was not described in such mutants. Therefore, other factors, in addition to MAG–ganglioside disruption, may be involved in NA-induced vacuolation. Furthermore, temporal factors should also be considered, as they might contribute to those differences: in Mag-null and *B4galnt1*-null mutants, the MAG–ganglioside interaction is permanently disrupted, while in NA-induced vacuolation, the disruption is transient. Similar intramyelinic vacuolation has been found in mitochondrial encephalopathies as Kearns–Sayre syndrome (60), in Canavan’s disease (61), in vitamin B12-deficient animals (62), in toxin-induced injuries (63), and in retroviral infections (64). Although in some of these myelinopathies the authors have placed the oligodendrocytes in the center of the disease (60, 65, 66), the mechanisms of vacuolation remain speculative in most of them.

The origin of several myelin pathologies, such as multiple sclerosis, acute disseminated encephalomyelitis, and acute hemorrhagic leukoencephalitis, has been related to neuroinflammatory processes (67). Mostly autoimmune demyelinating diseases are accompanied by inflammation of the CNS (67). The well-established notion that multiple sclerosis is an autoimmune and inflammatory disease has recently evolved toward the idea that it is a neurodegenerative disease with an inflammatory component (68, 69). On the other hand, chronic metabolic or mitochondrial encephalopathies are essentially devoid of neuroinflammatory component (70). In NA-injected rats, a neuroinflammatory process with an important infiltration of immunocompetent cells occurs (24). In order to explore whether the neuroinflammatory reaction induced by NA is the cause of myelin vacuolation, the anaphylatoxin C5a was ICV injected in rats. C5a is produced by the proteolysis of C5 component of complement and functions as an inflammatory signaling molecule, thereby inducing the activation of mast cells, basophils, and neutrophils which release histamine and pro-inflammatory cytokines (71). In addition, C5a displays a chemotactic function, thereby promoting the recruitment of neutrophils and macrophages to the inflammatory focus (72–74). In our study, ICV injection of C5a induced a neuroinflammatory reaction similar to that provoked by the ICV injection of NA. However, the vacuolation of axonal bundles observed in NA-treated rats was absent in C5a-injected rats, in spite of the evident neuroinflammatory reaction. These results suggest that the neuroinflammation process, including invasion of ventricles, periventricular areas, meninges, and choroid plexus by immunocompetent cells, is not the cause of the myelin vacuolation events observed in NA-injected rats.

The function of the complement system in myelinopathies (including demyelination) has been a matter of debate (26). While a protective role of complement in demyelination has been reported (27, 28), other authors suggest that the complement does not participate in demyelination (35), or have a role in immune-mediated myelin diseases (29–34). In the classical pathway of the complement, activated C1 induces the cleavage of C4 to form the anaphylatoxin C4a (75). Upregulation of C4A has been described in the CSF of a fulminant case of multiple sclerosis (76). In another study, two factors involved in complement activation, clusterin and C3, were identified in the CSF of several patients of multiple sclerosis (77).

To assess the role of the complement system, and specifically of MAC, in NA-induced myelin vacuolation, we pursued the immunolocalization of C5b9 on vacuolated myelin bundles and evaluated NA-induced vacuolation under situations of partial inhibition or complete blockade of MAC formation. Immunostaining with anti-C5b9 (MAC) did not label the damaged axonal bundles; however, MAC labeling was evident in damaged ependymal cells (25). Partial inhibition of MAC formation was achieved *in vivo* by blocking C5 activation with a monoclonal antibody administered systemically as well as ICV (25, 78, 79). C5-inhibited rats injected with NA displayed the same degree of myelin vacuolation as non-inhibited rats. The damage provoked by NA under C5 inhibition could have several explanations: (i) NA activates the complement system downstream of C5 in the proteolytic cascade; this is unlikely because *in vitro* experiments showed that NA-induced activation of complement at the level of C3 and generates iC3b in human serum (39); (ii) other mechanisms, independent of complement, could account for the vacuolation of the myelin. A second model of *in vivo* MAC deficiency is provided by C6 mutant rats, which bear a spontaneous deletion in the C6 gene (40, 80), preventing C6 synthesis and hence blocking formation of MAC and complement-mediated cell lysis (81, 82). In C6^−/−^ rats, the degree of myelin vacuolation after NA injection was similar to that of wild-type rats. Since MAC formation is completely precluded in C6 mutant rats (and was not detected by immunohistochemistry), these results lead us to conclude that the lytic pathway of complement does not participate in the myelin damage caused by NA.

Some viruses associated with subsequent myelin disorders contain NA in their coats, as is the case for influenza virus (83), parainfluenza virus (84), mumps virus (85), and measles virus (86). Acute disseminated encephalomyelitis and acute haemorrhagic leucoencephalitis are inflammatory demyelinating diseases that arise within 3 weeks of an infection with these viruses (87–90), among other viruses and bacteria (67). Subacute sclerosing panencephalitis, also called measles encephalitis, is a neuroinflammatory disease triggered by measles virus (91). Perivascular inflammation, gliosis in white matter, and irregular loss of myelin and axons in white matter are the hallmarks of this disease (67). The presence of NA in the virus coat confers a higher degree of virulence (92, 93). In the event of a brain infection by any of these NA-bearing viruses, NA could disturb the axon–myelin stability as seen in our NA-injected rats. Thus, NA might contribute to the myelin disorders associated with certain viral infections.

In summary, a single ICV injection of NA in rats induces a transient myelin vacuolation that consistently affects the SM, the optic chiasm, and the fimbria. The location of the vacuoles between the myelin sheath and the axonal plasma membrane suggests a disturbance of the interaction between the innermost wrap of myelin and the axon membrane. A possible disruption (provoked by NA) of the interaction between MAG and gangliosides at this level is hypothesized. The myelin damage observed seems to be moderate, as it reverts in 2 weeks, and is not accompanied by cell loss (the oligodendrocyte population within the affected tracts remains unaltered, and no neurodegeneration is detected in the related neuronal nuclei). The possibility that the cause of the vacuolation process could be the neuroinflammation occurring upon NA injection was ruled out based on the results obtained with the ICV injection of anaphylatoxin C5a. Finally, various forms of evidence lead us to discard the participation of the complement system in the process of vacuolation induced by NA. Therefore, the direct sialidase action of NA on myelin sheaths seems to be the most probable cause of vacuolation. The knowledge of the mechanisms underlying the myelin disorders caused by NA-bearing pathogens of the CNS may help to design more effective therapeutic strategies.

## Author Contributions

PG-D, ML-Á, BM, PF-L, and JG conceived and designed the study. PG-D, MC, MM, MF-A, and TH performed the experiments. KJ produced the anti-C5 Mab. PG-D, ML-Á, MC, MM, and PF-L analyzed the data. PG-D, ML-Á, and JG wrote the manuscript. All the authors read and approved the final manuscript.

## Conflict of Interest Statement

Competing interest statement of KJ: “I declare that I am a paid employee of Alexion Pharmaceuticals, Inc. and own shares of stock in Alexion Pharmaceuticals, Inc.” The other authors have declared that no other competing interests exist.

## References

[1] Duncan ID Radcliff AB. Inherited and acquired disorders of myelin: the underlying myelin pathology. Exp Neurol (2016) 283(Pt B):452–75. 10.1016/j.expneurol.2016.04.002 27068622 PMC5010953

[2] Walis A Liberski PP Brown P Ultrastructural alterations in the optic nerve in transmissible spongiform encephalopathies or prion diseases – a review. Folia Neuropathol (2004) 42(Suppl B):153–60. 16903149

[3] Liberski PP Spongiform change – an electron microscopic view. Folia Neuropathol (2004) 42(Suppl B):59–70. 16903142

[4] Wells GA Wilesmith JW McGill IS. Bovine spongiform encephalopathy: a neuropathological perspective. Brain Pathol (1991) 1(2):69–78. 10.1111/j.1750-3639.1991.tb00642.x 1688299

[5] Kreutzberg GW Blakemore WF Graeber MB Cellular pathology of the central nervous system. 6th ed In: Graham DI Lantos PL, editors. Greenfield’s Neuropathology. London: Arnold (1997). p. 85–140.

[6] Jubb KVF Huxtable CR The nervous system. 4th ed In: Jubb KVF Kennedy PC Palmer N, editors. Pathology of Domestic Animals. San Diego: Academic Press, Inc. (1993). p. 267–439.

[7] Bugiani M Postma N Polder E Dieleman N Scheffer PG Sim FJ Hyaluronan accumulation and arrested oligodendrocyte progenitor maturation in vanishing white matter disease. Brain (2013) 136(Pt 1):209–22. 10.1093/brain/aws320 23365098

[8] Matalon RM Michals-Matalon K Spongy degeneration of the brain, Canavan disease: biochemical and molecular findings. Front Biosci (2000) 5:D307–11. 10.2741/Matalon 10704428

[9] Greco CM Powell HC Garrett RS Lampert PW. Cycloleucine encephalopathy. Neuropathol Appl Neurobiol (1980) 6(5):349–60. 10.1111/j.1365-2990.1980.tb00671.x 6256679

[10] van der Lugt JJ Olivier J Jordaan P. Status spongiosis, optic neuropathy, and retinal degeneration in *Helichrysum argyrosphaerum* poisoning in sheep and a goat. Vet Pathol (1996) 33(5):495–502. 10.1177/030098589603300503 8885175

[11] van der Lugt JJ Venter I. Myelin vacuolation, optic neuropathy and retinal degeneration after closantel overdosage in sheep and in a goat. J Comp Pathol (2007) 136(2–3):87–95. 10.1016/j.jcpa.2006.11.007 17270202

[12] Ji J Yan X Li Z Lai Z Liu J. Therapeutic effects of intrathecal versus intravenous monosialoganglioside against bupivacaine-induced spinal neurotoxicity in rats. Biomed Pharmacother (2015) 69:311–6. 10.1016/j.biopha.2014.12.020 25661376

[13] DeMarco ML Woods RJ Atomic-resolution conformational analysis of the GM3 ganglioside in a lipid bilayer and its implications for ganglioside-protein recognition at membrane surfaces. Glycobiology (2009) 19(4):344–55. 10.1093/glycob/cwn137 19056784 PMC2733776

[14] Quarles RH. Myelin-associated glycoprotein (MAG): past, present and beyond. J Neurochem (2007) 100(6):1431–48. 10.1111/j.1471-4159.2006.04319.x 17241126

[15] Collins BE Yang LJ Mukhopadhyay G Filbin MT Kiso M Hasegawa A Sialic acid specificity of myelin-associated glycoprotein binding. J Biol Chem (1997) 272(2):1248–55. 10.1074/jbc.272.2.1248 8995428

[16] Strenge K Schauer R Bovin N Hasegawa A Ishida H Kiso M Glycan specificity of myelin-associated glycoprotein and sialoadhesin deduced from interactions with synthetic oligosaccharides. Eur J Biochem (1998) 258(2):677–85. 10.1046/j.1432-1327.1998.2580677.x 9874234

[17] Collins BE Ito H Sawada N Ishida H Kiso M Schnaar RL. Enhanced binding of the neural siglecs, myelin-associated glycoprotein and Schwann cell myelin protein, to Chol-1 (alpha-series) gangliosides and novel sulfated Chol-1 analogs. J Biol Chem (1999) 274(53):37637–43. 10.1074/jbc.274.53.37637 10608819

[18] Trapp BD Andrews SB Cootauco C Quarles R. The myelin-associated glycoprotein is enriched in multivesicular bodies and periaxonal membranes of actively myelinating oligodendrocytes. J Cell Biol (1989) 109(5):2417–26. 10.1083/jcb.109.5.2417 2478568 PMC2115868

[19] Schnaar RL. Brain gangliosides in axon-myelin stability and axon regeneration. FEBS Lett (2010) 584(9):1741–7. 10.1016/j.febslet.2009.10.011 19822144 PMC2856809

[20] Corfield AP Higa H Paulson JC Schauer R. The specificity of viral and bacterial sialidases for alpha(2-3)- and alpha(2-6)-linked sialic acids in glycoproteins. Biochim Biophys Acta (1983) 744(2):121–6. 10.1016/0167-4838(83)90080-8 6301560

[21] Gomez-Roldan MC Perez-Martin M Capilla-Gonzalez V Cifuentes M Perez J Garcia-Verdugo JM Neuroblast proliferation on the surface of the adult rat striatal wall after focal ependymal loss by intracerebroventricular injection of neuraminidase. J Comp Neurol (2008) 507(4):1571–87. 10.1002/cne.21618 18236450

[22] Pérez-Martín M Papel del epitelio ependimario en la estabilidad de los ventrículos cerebrales y en la neurogénesis en el adulto. Universidad de Málaga, Malaga (2000).

[23] Grondona JM Perez-Martin M Cifuentes M Perez J Jimenez AJ Perez-Figares JM Ependymal denudation, aqueductal obliteration and hydrocephalus after a single injection of neuraminidase into the lateral ventricle of adult rats. J Neuropathol Exp Neurol (1996) 55(9):999–1008. 10.1097/00005072-199609000-00007 8800096

[24] Granados-Duran P Lopez-Avalos MD Grondona JM Gomez-Roldan Mdel C Cifuentes M Perez-Martin M Neuroinflammation induced by intracerebroventricular injection of microbial neuraminidase. Front Med (2015) 2:14. 10.3389/fmed.2015.00014 25853134 PMC4362343

[25] Granados-Duran P Lopez-Avalos MD Hughes TR Johnson K Morgan BP Tamburini PP Complement system activation contributes to the ependymal damage induced by microbial neuraminidase. J Neuroinflammation (2016) 13(1):115. 10.1186/s12974-016-0576-9 27209022 PMC4875702

[26] Bergmann CC Lane TE Stohlman SA. Coronavirus infection of the central nervous system: host-virus stand-off. Nat Rev Microbiol (2006) 4(2):121–32. 10.1038/nrmicro1343 16415928 PMC7096820

[27] Tegla CA Cudrici C Rus V Ito T Vlaicu S Singh A Neuroprotective effects of the complement terminal pathway during demyelination: implications for oligodendrocyte survival. J Neuroimmunol (2009) 213(1–2):3–11. 10.1016/j.jneuroim.2009.06.006 19577811 PMC2756021

[28] Briggs DT Martin CB Ingersoll SA Barnum SR Martin BK. Astrocyte-specific expression of a soluble form of the murine complement control protein Crry confers demyelination protection in the cuprizone model. Glia (2007) 55(14):1405–15. 10.1002/glia.20551 17674370

[29] Dyer JK Bourque JA Steeves JD. The role of complement in immunological demyelination of the mammalian spinal cord. Spinal Cord (2005) 43(7):417–25. 10.1038/sj.sc.3101737 15897918

[30] Urich E Gutcher I Prinz M Becher B. Autoantibody-mediated demyelination depends on complement activation but not activatory Fc-receptors. Proc Natl Acad Sci U S A (2006) 103(49):18697–702. 10.1073/pnas.0607283103 17121989 PMC1693725

[31] Hundgeburth LC Wunsch M Rovituso D Recks MS Addicks K Lehmann PV The complement system contributes to the pathology of experimental autoimmune encephalomyelitis by triggering demyelination and modifying the antigen-specific T and B cell response. Clin Immunol (2013) 146(3):155–64. 10.1016/j.clim.2012.12.007 23352967

[32] Mead RJ Singhrao SK Neal JW Lassmann H Morgan BP. The membrane attack complex of complement causes severe demyelination associated with acute axonal injury. J Immunol (2002) 168(1):458–65. 10.4049/jimmunol.168.1.458 11751993

[33] Mead RJ Neal JW Griffiths MR Linington C Botto M Lassmann H Deficiency of the complement regulator CD59a enhances disease severity, demyelination and axonal injury in murine acute experimental allergic encephalomyelitis. Lab Invest (2004) 84(1):21–8. 10.1038/sj.labinvest.3700015 14631387

[34] Nataf S Carroll SL Wetsel RA Szalai AJ Barnum SR. Attenuation of experimental autoimmune demyelination in complement-deficient mice. J Immunol (2000) 165(10):5867–73. 10.4049/jimmunol.165.10.5867 11067947

[35] Brew BJ Davies NWS Cinque P Clifford DB Nath A. Progressive multifocal leukoencephalopathy and other forms of JC virus disease. Nat Rev Neurol (2010) 6(12):667–79. 10.1038/nrneurol.2010.164 21131916

[36] Kurosawa K Misu T Takai Y Sato DK Takahashi T Abe Y Severely exacerbated neuromyelitis optica rat model with extensive astrocytopathy by high affinity anti-aquaporin-4 monoclonal antibody. Acta Neuropathol Commun (2015) 3:82. 10.1186/s40478-015-0259-2 26637322 PMC4670539

[37] Pangburn MK Pangburn KL Koistinen V Meri S Sharma AK Molecular mechanisms of target recognition in an innate immune system: interactions among factor H, C3b, and target in the alternative pathway of human complement. J Immunol (2000) 164(9):4742–51. 10.4049/jimmunol.164.9.4742 10779780

[38] Donin N Jurianz K Ziporen L Schultz S Kirschfink M Fishelson Z. Complement resistance of human carcinoma cells depends on membrane regulatory proteins, protein kinases and sialic acid. Clin Exp Immunol (2003) 131(2):254–63. 10.1046/j.1365-2249.2003.02066.x 12562385 PMC1808622

[39] Fujita T Ohi H Endo M Ohsawa I Kanmatsuse K. The role of sialidase in the development of hypocomplementemia in postinfectious acute glomerulonephritis. Clin Immunol (1999) 92(1):97–102. 10.1006/clim.1999.4729 10413657

[40] Ramaglia V King RH Nourallah M Wolterman R de Jonge R Ramkema M The membrane attack complex of the complement system is essential for rapid Wallerian degeneration. J Neurosci (2007) 27(29):7663–72. 10.1523/JNEUROSCI.5623-06.2007 17634361 PMC6672891

[41] Casarsa C De Luigi A Pausa M De Simoni MG Tedesco F. Intracerebroventricular injection of the terminal complement complex causes inflammatory reaction in the rat brain. Eur J Immunol (2003) 33(5):1260–70. 10.1002/eji.200323574 12731051

[42] Rother RP Arp J Jiang J Ge W Faas SJ Liu W C5 blockade with conventional immunosuppression induces long-term graft survival in presensitized recipients. Am J Transplant (2008) 8(6):1129–42. 10.1111/j.1600-6143.2008.02222.x 18444931

[43] Rollins SA Matis LA Springhorn JP Setter E Wolff DW. Monoclonal antibodies directed against human C5 and C8 block complement-mediated damage of xenogeneic cells and organs. Transplantation (1995) 60(11):1284–92. 10.1097/00007890-199512000-00017 8525523

[44] Costa C Zhao L Shen Y Su X Hao L Colgan SP Role of complement component C5 in cerebral ischemia/reperfusion injury. Brain Res (2006) 1100(1):142–51. 10.1016/j.brainres.2006.05.029 16780818

[45] Ortiz-Hidalgo C Bouin PA Bouin’s fixative and other contributions to medicine. Arch Pathol Lab Med (1992) 116(8):882–4. 1497471

[46] Karnovsky MJ A formaldehyde-glutaraldehyde fixative of high osmolality for use in electron microscopy. J Cell Biol (1965) 27:137–8.

[47] Schmued LC Hopkins KJ. Fluoro-Jade B: a high affinity fluorescent marker for the localization of neuronal degeneration. Brain Res (2000) 874(2):123–30. 10.1016/S0006-8993(00)02513-0 10960596

[48] Marie Y Sanson M Mokhtari K Leuraud P Kujas M Delattre JY OLIG2 as a specific marker of oligodendroglial tumour cells. Lancet (2001) 358(9278):298–300. 10.1016/s0140-6736(01)05499-x 11498220

[49] Granados-Duran P Análisis de la reacción inflamatoria y los procesos degenerativos provocados por la inyección intracerebroventricular de neuraminidasa en rata. University of Malaga, Malaga (2014).

[50] Crocker PR Clark EA Filbin M Gordon S Jones Y Kehrl JH Siglecs: a family of sialic-acid binding lectins. Glycobiology (1998) 8(2):v 10.1093/oxfordjournals.glycob.a018832 9498912

[51] Kelm S Pelz A Schauer R Filbin MT Tang S de Bellard ME Sialoadhesin, myelin-associated glycoprotein and CD22 define a new family of sialic acid-dependent adhesion molecules of the immunoglobulin superfamily. Curr Biol (1994) 4(11):965–72. 10.1016/S0960-9822(00)00220-7 7533044

[52] Trapp BD Myelin-associated glycoprotein. Location and potential functions. Ann N Y Acad Sci (1990) 605:29–43. 10.1111/j.1749-6632.1990.tb42378.x 1702602

[53] Sturgill ER Aoki K Lopez PH Colacurcio D Vajn K Lorenzini I Biosynthesis of the major brain gangliosides GD1a and GT1b. Glycobiology (2012) 22(10):1289–301. 10.1093/glycob/cws103 22735313 PMC3425327

[54] Yang LJ Zeller CB Shaper NL Kiso M Hasegawa A Shapiro RE Gangliosides are neuronal ligands for myelin-associated glycoprotein. Proc Natl Acad Sci U S A (1996) 93(2):814–8. 10.1073/pnas.93.2.814 8570640 PMC40139

[55] Vyas AA Patel HV Fromholt SE Heffer-Lauc M Vyas KA Dang J Gangliosides are functional nerve cell ligands for myelin-associated glycoprotein (MAG), an inhibitor of nerve regeneration. Proc Natl Acad Sci U S A (2002) 99(12):8412–7. 10.1073/pnas.072211699 12060784 PMC123081

[56] Fruttiger M Montag D Schachner M Martini R. Crucial role for the myelin-associated glycoprotein in the maintenance of axon-myelin integrity. Eur J Neurosci (1995) 7(3):511–5. 10.1111/j.1460-9568.1995.tb00347.x 7539694

[57] Pernet V Joly S Christ F Dimou L Schwab ME. Nogo-A and myelin-associated glycoprotein differently regulate oligodendrocyte maturation and myelin formation. J Neurosci (2008) 28(29):7435–44. 10.1523/JNEUROSCI.0727-08.2008 18632947 PMC6670388

[58] Banizs B Pike MM Millican CL Ferguson WB Komlosi P Sheetz J Dysfunctional cilia lead to altered ependyma and choroid plexus function, and result in the formation of hydrocephalus. Development (2005) 132(23):5329–39. 10.1242/dev.02153 16284123

[59] Susuki K Baba H Tohyama K Kanai K Kuwabara S Hirata K Gangliosides contribute to stability of paranodal junctions and ion channel clusters in myelinated nerve fibers. Glia (2007) 55(7):746–57. 10.1002/glia.20503 17352383

[60] Szalardy L Molnar M Torok R Zadori D Vecsei L Klivenyi P Histopathological comparison of Kearns-Sayre syndrome and PGC-1alpha-deficient mice suggests a novel concept for vacuole formation in mitochondrial encephalopathy. Folia Neuropathol (2016) 54(1):9–22. 10.5114/fn.2016.58911 27179217

[61] Roscoe RB Elliott C Zarros A Baillie GS. Non-genetic therapeutic approaches to Canavan disease. J Neurol Sci (2016) 366:116–24. 10.1016/j.jns.2016.05.012 27288788

[62] Agamanolis DP Victor M Harris JW Hines JD Chester EM Kark JA. An ultrastructural study of subacute combined degeneration of the spinal cord in vitamin B12-deficient rhesus monkeys. J Neuropathol Exp Neurol (1978) 37(3):273–99. 10.1097/00005072-197805000-00006 96220

[63] Love S. Cuprizone neurotoxicity in the rat: morphologic observations. J Neurol Sci (1988) 84(2–3):223–37. 10.1016/0022-510X(88)90127-X 2837540

[64] Bergmann M Gullotta F Kuchelmeister K Masini T Angeli G. AIDS-myelopathy. A neuropathological study. Pathol Res Pract (1993) 189(1):58–65. 10.1016/S0344-0338(11)80117-2 8516218 PMC7130719

[65] Francis JS Markov V Leone P. Dietary triheptanoin rescues oligodendrocyte loss, dysmyelination and motor function in the nur7 mouse model of Canavan disease. J Inherit Metab Dis (2014) 37(3):369–81. 10.1007/s10545-013-9663-6 24288037

[66] Blakemore WF Observations on oligodendrocyte degeneration, the resolution of status spongiosus and remyelination in cuprizone intoxication in mice. J Neurocytol (1972) 1(4):413–26. 10.1007/BF01102943 8530973

[67] Love S. Demyelinating diseases. J Clin Pathol (2006) 59(11):1151–9. 10.1136/jcp.2005.031195 17071802 PMC1860500

[68] Stys PK Zamponi GW van Minnen J Geurts JJ. Will the real multiple sclerosis please stand up? Nat Rev Neurosci (2012) 13(7):507–14. 10.1038/nrn3275 22714021

[69] Trapp BD Nave KA. Multiple sclerosis: an immune or neurodegenerative disorder? Annu Rev Neurosci (2008) 31:247–69. 10.1146/annurev.neuro.30.051606.094313 18558855

[70] Ellison D Love S Chimelli LMC Harding B Lowe JS Vinters HV Neuropathology: A Reference Text of CNS Pathology. 3rd ed Edinburgh: Mosby Ltd (2012).

[71] Klos A Tenner AJ Johswich K-O Ager RR Reis ES Köhl J. The role of the anaphylatoxins in health and disease. Mol Immunol (2009) 46(14):2753–66. 10.1016/j.molimm.2009.04.027 19477527 PMC2725201

[72] Wetsel RA. Structure, function and cellular expression of complement anaphylatoxin receptors. Curr Opin Immunol (1995) 7(1):48–53. 10.1016/0952-7915(95)80028-X 7772282

[73] Isfort K Ebert F Bornhorst J Sargin S Kardakaris R Pasparakis M Real-time imaging reveals that P2Y2 and P2Y12 receptor agonists are not chemoattractants and macrophage chemotaxis to complement C5a is phosphatidylinositol 3-kinase (PI3K)- and p38 mitogen-activated protein kinase (MAPK)-independent. J Biol Chem (2011) 286(52):44776–87. 10.1074/jbc.M111.289793 22057273 PMC3247992

[74] Webster RO Zanolari B Henson PM Neutrophil chemotaxis in response to surface-bound C5A. Exp Cell Res (1980) 129(1):55–62. 10.1016/0014-4827(80)90330-4 7428814

[75] Langone JJ Das C Bennett D Terman DS. Generation of human C3a, C4a, and C5a anaphylatoxins by protein A of *Staphylococcus aureus* and immobilized protein A reagents used in serotherapy of cancer. J Immunol (1984) 133(2):1057–63. 6610702

[76] Fuvesi J Hanrieder J Bencsik K Rajda C Kovacs SK Kaizer L Proteomic analysis of cerebrospinal fluid in a fulminant case of multiple sclerosis. Int J Mol Sci (2012) 13(6):7676–93. 10.3390/ijms13067676 22837721 PMC3397553

[77] Stoop MP Dekker LJ Titulaer MK Burgers PC Sillevis Smitt PA Luider TM Multiple sclerosis-related proteins identified in cerebrospinal fluid by advanced mass spectrometry. Proteomics (2008) 8(8):1576–85. 10.1002/pmic.200700446 18351689

[78] Vakeva A Meri S. Complement activation and regulator expression after anoxic injury of human endothelial cells. APMIS (1998) 106(12):1149–56. 10.1111/j.1699-0463.1998.tb00271.x 10052723

[79] Peckham RM Handrigan MT Bentley TB Falabella MJ Chrovian AD Stahl GL C5-blocking antibody reduces fluid requirements and improves responsiveness to fluid infusion in hemorrhagic shock managed with hypotensive resuscitation. J Appl Physiol (2007) 102(2):673–80. 10.1152/japplphysiol.00917.2006 17068213

[80] Bhole D Stahl GL. Molecular basis for complement component 6 (C6) deficiency in rats and mice. Immunobiology (2004) 209(7):559–68. 10.1016/j.imbio.2004.08.001 15568620

[81] Leenaerts PL Stad RK Hall BM Van Damme BJ Vanrenterghem Y Daha MR. Hereditary C6 deficiency in a strain of PVG/c rats. Clin Exp Immunol (1994) 97(3):478–82. 10.1111/j.1365-2249.1994.tb06113.x 8082303 PMC1534854

[82] van Dixhoorn MG Timmerman JJ Van Gijlswijk-Janssen DJ Muizert Y Verweij C Discipio RG Characterization of complement C6 deficiency in a PVG/c rat strain. Clin Exp Immunol (1997) 109(2):387–96. 10.1046/j.1365-2249.1997.4551354.x 9276537 PMC1904735

[83] Seto JBR Rott R Isolation of a low molecular weight sialidase (neuraminidase) from influenza virus. Biochim Biophys Acta (1966) 113(2):402–4. 10.1016/S0926-6593(66)80081-4 5942440

[84] Shibuta H Nozawa A Shioda T Kanda T. Neuraminidase activity and syncytial formation in variants of parainfluenza 3 virus. Infect Immun (1983) 41(2):780–8. 6307881 10.1128/iai.41.2.780-788.1983 PMC264708

[85] Klamm H. Some properties of mumps virus neuraminidase. Acta Virol (1980) 24(2):127–31. 6158851

[86] Howe C Newcomb EW Lee LT The neuraminidase of measles virus. Biochem Biophys Res Commun (1969) 34(4):388–91. 10.1016/0006-291X(69)90393-3 5813332

[87] Voudris KA Vagiakou EA Skardoutsou A. Acute disseminated encephalomyelitis associated with parainfluenza virus infection of childhood. Brain Dev (2002) 24(2):112–4. 10.1016/S0387-7604(02)00008-6 11891105

[88] Ozkale Y Erol I Ozkale M Demir S Alehan F. Acute disseminated encephalomyelitis associated with influenza A H1N1 infection. Pediatr Neurol (2012) 47(1):62–4. 10.1016/j.pediatrneurol.2012.03.019 22704021 PMC7127454

[89] Nardone R Golaszewski S Trinka E Tezzon F Zuccoli G. Acute disseminated encephalomyelitis preceding measles exanthema. J Child Neurol (2011) 26(12):1590–2. 10.1177/0883073811413130 21771949

[90] Sonmez FM Odemis E Ahmetoglu A Ayvaz A. Brainstem encephalitis and acute disseminated encephalomyelitis following mumps. Pediatr Neurol (2004) 30(2):132–4. 10.1016/j.pediatrneurol.2003.09.004 14984908

[91] Johannes RS Sever JL Subacute sclerosing panencephalitis. Annu Rev Med (1975) 26:589–601. 10.1146/annurev.me.26.020175.003105 1096780

[92] Malik T Wolbert C Mauldin J Sauder C Carbone KM Rubin SA. Functional consequences of attenuating mutations in the haemagglutinin neuraminidase, fusion and polymerase proteins of a wild-type mumps virus strain. J Gen Virol (2007) 88(Pt 9):2533–41. 10.1099/vir.0.82935-0 17698664

[93] Bhatia A Kast RE. How influenza’s neuraminidase promotes virulence and creates localized lung mucosa immunodeficiency. Cell Mol Biol Lett (2007) 12(1):111–9. 10.2478/s11658-006-0055-x 17103087 PMC6275963

